# Case Report: Longitudinal evaluation of cerebrospinal fluid Alzheimer’s disease core biomarkers in a case of refractory late-onset epilepsy of unknown etiology

**DOI:** 10.3389/fnagi.2026.1807027

**Published:** 2026-10-12

**Authors:** Takafumi Wada, Akira Kuzuya, Kazuma Koda, Sakiho Ueda, Atsushi Shima, Nobukatsu Sawamoto, Akio Ikeda, Ryosuke Takahashi, Riki Matsumoto

**Affiliations:** 1 Department of Neurology, Kyoto University Graduate School of Medicine, Kyoto, Japan; 2 Department of Human Health Science, Kyoto University Graduate School of Medicine, Kyoto, Japan; 3 Department of Epilepsy, Movement Disorders, and Physiology, Kyoto University Graduate School of Medicine, Kyoto, Japan; 4 Office of Research Acceleration, Kyoto University, Kyoto, Japan

**Keywords:** Alzheimer’s disease, amyloid PET, cerebrospinal fluid AD biomarkers, electroencephalography, late-onset epilepsy of unknown etiology

## Abstract

In recent years, late-onset epilepsy of unknown etiology (LOEU) has increasingly been recognized as a risk factor for Alzheimer’s disease (AD) and other forms of dementia. We present a case of refractory LOEU with progressive cognitive decline, longitudinally evaluated over more than 20 years using neuropsychological tests, cerebrospinal fluid (CSF) AD core biomarkers (Aβ42/40 ratio, p-tau), electroencephalography, and neuroimaging. The CSF AD core biomarkers were consistently positive, indicating the presence of both amyloid and tau pathology; however, CSF Aβ levels markedly decreased in parallel with improved seizure control. Notably, the addition of low-dose perampanel (1 mg/day) led to improvement in both cognitive function and seizure control, while ^18^F-flutemetamol positron emission tomography remained visually negative despite exceeding the predefined quantitative Centiloid threshold. Two years later, seizure frequency increased again, cognitive function deteriorated, and amyloid PET became visually positive, accompanied by an increase in the quantitative Centiloid value. This longitudinal observation suggests that chronic epileptic activity may modify or confound the interpretation of CSF AD biomarker profiles and contribute to cognitive decline, while concomitant early or slowly progressive AD pathology cannot be excluded. The overall biomarker trajectory, including persistently positive CSF AD biomarkers and progressive amyloid PET findings, is compatible with evolving AD pathology, although the marked decline in absolute CSF Aβ concentrations during improved seizure control suggests that chronic epileptic activity may also influence biomarker dynamics. Careful interpretation of AD biomarkers is therefore essential in patients with LOEU, particularly in the era of anti-amyloid therapies.

## Introduction

In Alzheimer’s disease (AD), focal impaired awareness seizures (FIAS) or subclinical seizures without obvious clinical manifestations are frequently observed in both the early and late stages of the disease. Pathological studies have also revealed the presence of amyloid-β (Aβ) pathology (senile plaques) and phosphorylated tau pathology (neurofibrillary tangles) in the patients with refractory epilepsy, suggesting that epilepsy may accelerate AD-related pathological processes (Mackenzie et al., 1996; Tai et al., 2016; Gourmaud et al., 2020). These findings indicate a shared underlying pathophysiology between AD and epilepsy.

In recent years, the incidence of new-onset epilepsy in adults aged ≥55 years has increased (Beghi and Giussani, 2018; Hernández-Ronquillo et al., 2018). Late-onset epilepsy of unknown etiology (LOEU), defined by the absence of structural abnormalities on neuroimaging such as brain magnetic resonance imaging (MRI), is now recognized as a potential risk factor for AD and other forms of dementia (Ophir et al., 2021). In the context of recent advances in anti-amyloid antibody therapies, accurate diagnosis of AD based on biomarker assessments within an appropriate clinical context and appropriate selection of patients eligible for disease-modifying treatments have become increasingly important (Ramanan et al., 2023). To achieve this goal, it is crucial to discriminate between AD with epilepsy and LOEU with cognitive decline. However, detailed longitudinal reports of LOEU with extended follow-up remain scarce. Here, we present a case of LOEU that was longitudinally followed for more than 20 years.

## Case description

A right-handed man with 16 years of education presented to our hospital at the age of 60 with episodes of transient forgetfulness. He had no relevant medical history or comorbidities associated with his symptoms. A schematic overview of the clinical course is shown in Figure 1A. One year later, he developed FIAS characterized by blank staring and oral automatism preceded by olfactory hallucinations. Although routine electroencephalography (EEG) did not reveal apparent epileptiform activity, he was clinically diagnosed with medial temporal lobe epilepsy and initially treated with carbamazepine. Owing to a drug eruption, treatment was switched to valproic acid monotherapy up to 800 mg/day, resulting in seizure remission by the age of 64.

**Figure 1:**
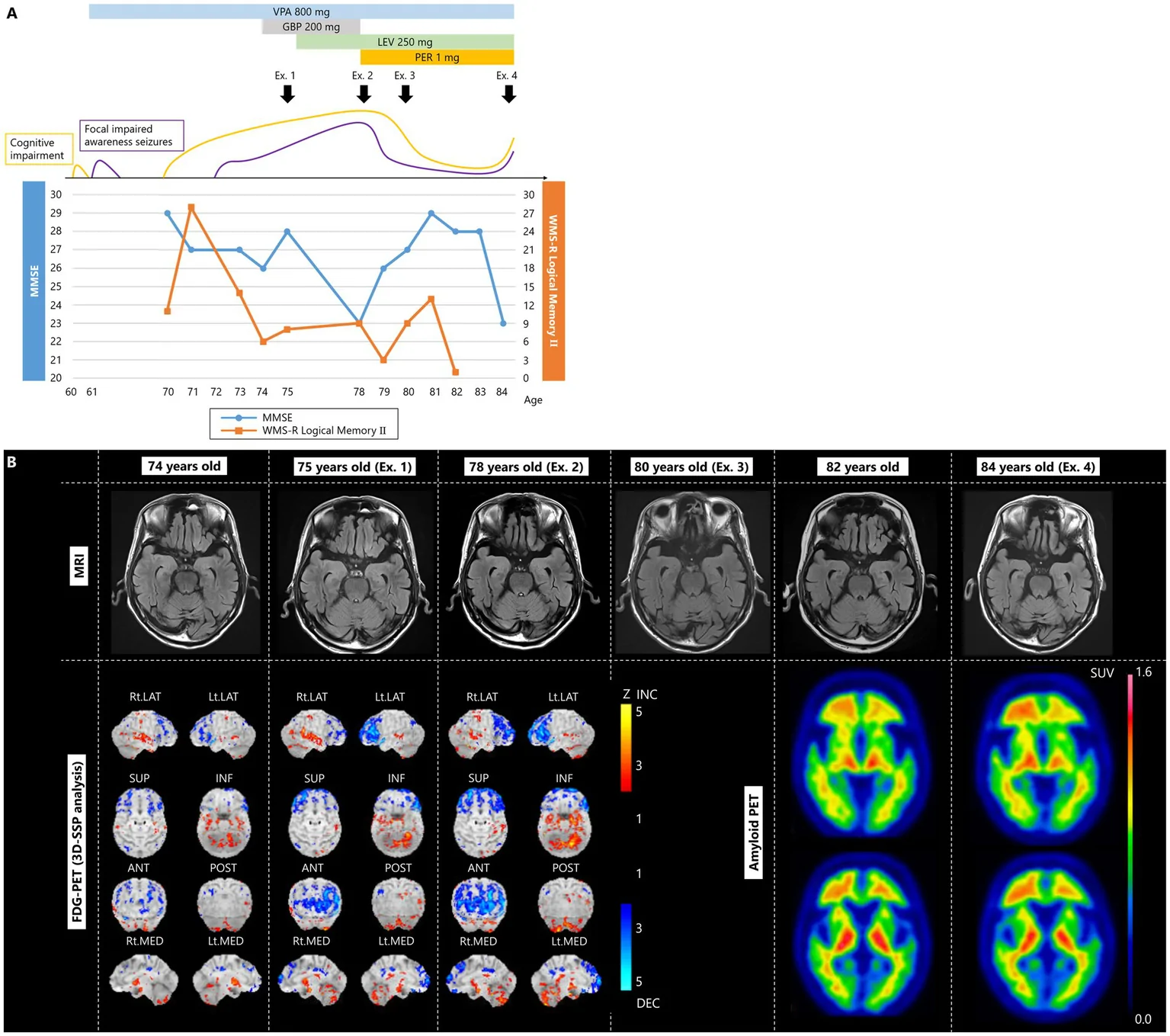
**(A)** Schematic representation of the clinical course. Following the addition of perampanel at a dose of 1 mg/day, seizure frequency decreased and cognitive functions improved to levels comparable to those observed at age 70. **(B)** Imaging findings at ages 74, 75, 78, 80, 82, and 84 years. The upper row shows brain magnetic resonance imaging (MRI), the lower row shows three-dimensional stereotactic surface projection (3D-SSP) analysis of ^18^F-fluorodeoxyglucose-positron emission tomography (FDG-PET), in which color-coded maps represent *Z*-scores indicating the degree of regional hypermetabolism or hypometabolism compared with age-matched normal controls, and the ^18^F-flutemetamol PET (amyloid PET). MRI revealed mild diffuse cerebral atrophy at all examined ages. FDG-PET showed no apparent hypometabolism at age 74. At age 75, FDG-PET showed no significant change compared with the previous year; however, hypometabolism in the bilateral frontal lobes became evident at age 78. Amyloid PET at age 82 showed no definite cortical tracer uptake on visual assessment, despite a Centiloid value of 26.9, whereas cortical tracer uptake in the right frontal cortex was clearly observed on visual assessment at age 84 (Centiloid value: 37.9). VPA, valproic acid; GBP, gabapentin; LEV, levetiracetam; PER, perampanel; MMSE, Mini-Mental State Examination; WMS-R, Wechsler Memory Scale-Revised; MRI, magnetic resonance imaging; PET, positron emission tomography; FDG, ^18^F-fluorodeoxyglucose; 3D-SSP, three-dimensional stereotactic surface projection; SUV, standardized uptake value.

At the age of 70, the patient became aware of mild memory impairment. His Mini-Mental State Examination (MMSE) score was 29, and the delayed recall score of Logical Memory II in the Wechsler Memory Scale-Revised (WMS-R) was 11 (out of 50). Over time, his forgetfulness gradually worsened, accompanied by a decline in WMS-R logical memory performance. FIAS recurred at the age of 72, and seizure frequency further increased by the age of 74, at which point the delayed recall score declined to 6. Brain MRI, including susceptibility-weighted imaging sequences, revealed mild diffuse cerebral atrophy without hippocampal sclerosis, microbleeds, features suggestive of cerebral amyloid angiopathy, or significant vascular burden, while ^18^F-fluorodeoxyglucose positron emission tomography (FDG-PET) showed no significant hypometabolism (Figure 1B). As repeated EEG recordings demonstrated sharp waves in the right frontotemporal region, gabapentin was added for seizure management.

Despite treatment, cognitive dysfunction continued to worsen, and FIAS escalated to a daily occurrence. At the age of 75, the patient was admitted for comprehensive evaluation (Ex. 1 in Figure 1 and Table 1). Follow-up brain MRI and FDG-PET revealed no significant interval changes. During 24-h video EEG monitoring, six epileptiform discharges with corresponding clinical seizures were recorded, arising from the left fronto-temporal region and spreading across the left hemisphere (Figure 2). Cerebrospinal fluid (CSF) analysis revealed Aβ40 of 13,671 pg/mL, Aβ42 of 1,549 pg/mL (cutoff >674 pg/mL), an Aβ42/40 ratio of 0.113 (cutoff >0.118), and phosphorylated tau (p-tau) of 74.6 pg/mL (cutoff <50.0 pg/mL).

**Table 1:** Video electroencephalography findings and cerebrospinal fluid Alzheimer’s disease biomarkers at the ages of 75, 78, 80, and 84.

Parameter		Ex. 1 (75 years old)	Ex. 2 (78 years old)	Ex. 3 (80 years old)	Ex. 4 (84 years old)
Cognitive decline status		Chronic progression	Peak	Remission	Relapse
24-h video EEG	Ictal pattern	Arising from lt. fronto-temporal region 6 times/24 hours Average duration time is 23.75 seconds	Arising from rt. fronto-temporal region 4 times/24 hours Average duration time is 20 seconds	No clinical seizures	No clinical seizures
	Spike/Sharp wave (Awake)	1.28 discharges/hour at lt > rt. fronto-temporal region	0.14 discharges/hour at lt. fronto-temporal region	0.09 discharges/hour at lt. fronto-temporal region	0.52 discharges/hour at lt. fronto-temporal region
	Spike/Sharp wave (NREM)	0.46 discharges/hour at lt > rt. fronto-temporal region	0.97 discharges/hour at lt. fronto-temporal region	0.62 discharges/hour at lt. fronto-temporal region	0.96 discharges/hour at lt. fronto-temporal region
	Sharp transients (Awake)	8.11 discharges/hour at lt > rt. fronto-temporal region	0.53 discharges/hour at lt. fronto-temporal region	0.38 discharges/hour at lt. fronto-temporal region	1.73 discharges/hour at lt. fronto-temporal region
	Sharp transients (NREM)	23.90 discharges/hour at lt > rt. fronto-temporal region	0.78 discharges/hour at lt. fronto-temporal region	0.62 discharges/hour at lt. fronto-temporal region	2.99 discharges/hour at lt. fronto-temporal region
CSF biomarkers	Aβ40 (pg/mL)	13,671	13,050	8,214	N. A.
	Aβ42 (pg/mL) Cutoff: 674 pg/mL	1,549	1,196	720	N. A.
	Aβ42/40 ratio Cutoff: 0.118	0.113	0.092	0.088	N. A.
	p-tau (pg/mL) Cutoff: 50 pg/mL	74.6	74.6	80.9	N. A.

Sharp transients were not considered definitively epileptiform and were of uncertain epileptiform significance. EEG, electroencephalography; NREM, non-rapid eye movement sleep; CSF, cerebrospinal fluid; AD, Alzheimer’s disease. Ex., examination; N.A., not available.

**Figure 2:**
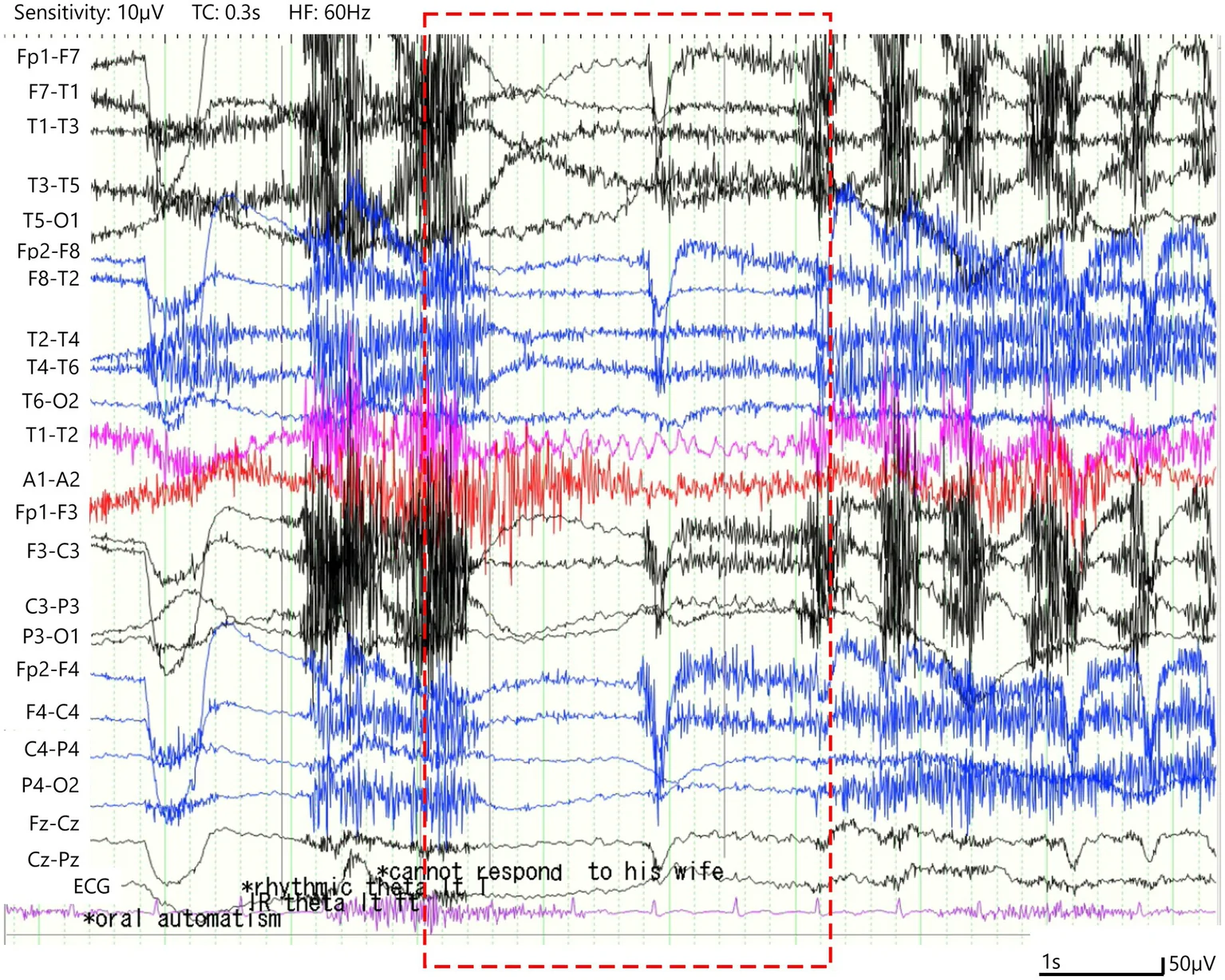
Representative video electroencephalography findings during a focal impaired awareness seizure at age 75. The ictal pattern is highlighted by the dashed box. TC, time constant; HF, high cut filter.

Levetiracetam was subsequently added; however, seizure control remained poor, leading to readmission at the age of 78 (Ex. 2 in Figure 1 and Table 1). MRI showed no significant changes, whereas FDG-PET demonstrated hypometabolism in the bilateral frontal lobes. During 24-h video EEG monitoring, four ictal epileptiform discharges arising from the right fronto-temporal region were observed along with interictal spikes and sharp waves in the left fronto-temporal region. CSF analysis revealed stable Aβ40 levels (13,050 pg/mL), but further reductions in Aβ42 levels (1,196 pg/mL) and the Aβ42/40 ratio (0.092), while p-tau level remained unchanged (74.6 pg/mL).

To exclude autoimmune encephalitis as a cause of refractory multifocal seizures despite three different antiseizure medications, a comprehensive CSF autoimmune antibody panel was performed. All tested antibodies—including anti-LGI1, anti-GAD, anti-NMDAR, anti-AMPAR, anti-CASPR2, anti-DNER, anti-DPPX, anti-GABAA receptor, anti-GABAB receptor, anti-mGluR1, anti-mGluR5, anti-IgLON5, anti-Homer3, anti-Hu, anti-Yo, anti-Ri, anti-Ma, anti-CV2, anti-amphiphysin antibodies—were negative. Gabapentin was then replaced with perampanel at a dose of 1 mg/day. Remarkably, seizure frequency was drastically reduced, although seizures did not completely resolve, following the introduction of extremely low-dose perampanel (Figure 1A), accompanied by improvements in MMSE and WMS-R logical memory scores.

Two years after the second admission, at the age of 80, MRI showed no progression of cerebral atrophy (Ex. 3 in Figure 1B). No ictal epileptiform discharges were detected on 24-h video EEG. CSF levels of Aβ40 and Aβ42 decreased (8,214 pg/mL and 720 pg/mL, respectively), while the Aβ42/40 ratio remained stable (0.088). The p-tau level was 80.9 pg/mL, comparable to previous measurements (Ex. 3 in Table 1).

At the age of 81, both the MMSE and WMS-R logical memory scores showed improvement, reaching levels comparable to those observed at the age of 70. The patient no longer met the diagnostic criteria for dementia, maintaining independence in activities of daily living. At the age of 82, brain MRI showed no significant changes compared with that obtained at the age of 78, and ^18^F-flutemetamol PET (amyloid PET) was visually assessed as negative for cortical amyloid deposition. However, semi-quantitative analysis showed a Centiloid value of 26.9, above the quantitative cutoff of 12.4 described in a previous study (Imabayashi et al., 2022) and a composite SUVR of 0.57, above the quantitative cutoff of 0.54 (Figure 1B). However, at the age of 84, seizure frequency increased and cognitive function deteriorated once more. Although no clinical seizures were recorded during 24-h video EEG, interictal epileptiform discharges were detected in the left fronto-temporal region (Ex. 4 in Table 1). The MMSE score declined to 23, and amyloid PET clearly demonstrated cortical tracer uptake in the right frontal cortex on visual assessment, and the Centiloid value increased to 37.9 and SUVR to 0.61 (Ex. 4 in Figure 1).

CSF samples were collected in sterile polypropylene tubes and were centrifuged for 10 min at 2,000 rpm, and aliquots were stored at −80 °C. All samples were measured after a single freeze–thaw cycle. Aβ40 and Aβ42 levels were measured using the human/rat Aβ40 ELISA kit (Wako, Tokyo, Japan), and the human/rat Aβ42 ELISA kit (Wako), respectively. All Aβ measurements were performed in duplicate, and all CSF samples were analysed simultaneously in a single analytical batch on the same ELISA plate after storage under identical conditions. CSF p-tau levels were measured by LSI Medience Corporation (Tokyo, Japan) using enzyme immunoassay as part of routine clinical practice at the time of each lumbar puncture.

In the present case, a 20-min list-mode PET scan was performed 90 min after the injection of 18F-flutemetamol (185 MBq; Vizamyl, Nihon Medi-Physics Co., Tokyo, Japan), consistent with the standard uptake window for this tracer using a PET/CT scanner (Discovery IQ; GE Healthcare, Milwaukee, WI, USA). Semi-quantitative amyloid PET analysis was performed using VIZCalc software (Nihon Medi-Physics Co., Ltd., Tokyo, Japan), based on the registration method without MRI as described in the previous research (Imabayashi et al., 2022). Partial-volume correction was not applied. Centiloid values were calculated with the voxels of interests provided in the Centiloid project (Klunk et al., 2015), using the whole cerebellum as the reference region. Both amyloid PET scans were quantified under identical conditions (the same processing pipeline, reference region, and acquisition time window) and Centiloid values were recomputed for both time points to ensure that the two measurements were directly comparable. A Centiloid value of 12.4, considered the optimal threshold for discriminating visually positive from visually negative scans, was used as the predefined quantitative reference. Composite Standardized Uptake Value Ratios (SUVRs) were calculated separately using multiple reference regions (whole cerebellum, pons, cerebellar gray matter, and whole cerebellum plus brainstem), and are presented in Supplementary Table 2. Two neuroradiologists performed visual interpretation and were not blinded to the quantitative results. When visual interpretation and quantitative assessment were discordant, both findings were reported explicitly.

## Discussion

This case study describes a patient with LOEU whose cognitive function, CSF AD core biomarkers, EEG findings, and neuroimaging results were longitudinally monitored over a 20-year period. CSF AD core biomarkers consistently showed reduced Aβ42/40 ratios and elevated p-tau levels throughout the clinical course. In contrast, amyloid PET demonstrated no apparent cortical amyloid deposition until a later stage of the disease. Notably, cognitive impairment in this patient was reversible and improved in parallel with seizure control following add-on treatment with perampanel.

A distinctive feature of the present case was the pattern of cerebral hypometabolism which predominantly involved the bilateral frontal lobes rather than the posterior cingulate gyrus, the precuneus, or the medial temporal lobes, regions typically affected in AD. Similar imaging patterns have been reported in cases of persistent epileptic amnesia (Ito et al., 2009) and transient epileptic amnesia, in which hypometabolism was observed in the frontal and temporal lobes (Mosbah et al., 2014). Frequent epileptic seizures are known to induce hypometabolism beyond the seizure focus, reflecting widespread network dysfunction (Merlet et al., 1996). In temporal lobe epilepsy, FDG-PET studies have demonstrated hypometabolism attributable to disrupted connectivity between the parahippocampal gyrus and the ipsilateral prefrontal cortex (Takaya et al., 2006). In the present case, excessive epileptic activity arising from the bilateral fronto-temporal regions was therefore considered responsible for the observed frontal hypometabolism.

Aβ monomers are produced and released into the interstitial fluid in response to neuronal excitation (Selkoe, 2006). A portion of interstitial fluid Aβ subsequently enters the CSF. In AD, preferential aggregation and deposition of Aβ42, rather than Aβ40, results in decreased CSF Aβ42 levels and a reduced Aβ42/40 ratio. Experimental studies using AD mouse models have demonstrated an initial increase in CSF Aβ levels, followed by subsequent decline as amyloid deposition progresses (Maia et al., 2015). Similarly, in familial AD, CSF Aβ levels are known to increase during the preclinical phase before decreasing with disease progression (Fagan et al., 2014). These observations suggest that transient elevation in CSF Aβ may reflect enhanced Aβ production preceding aggregation and deposition, which can later be detected by amyloid PET imaging. Furthermore, both CSF AD core biomarkers and amyloid PET abnormalities may precede the clinical onset of AD by more than 20 years (Jack Jr et al., 2013).

In the present case, elevated CSF Aβ levels were observed from the initial evaluation, accompanied by a reduced Aβ42/40 ratio despite preserved absolute CSF Aβ42 concentrations during periods of poor seizure control. During this phase, progressive cognitive decline was compatible with possible underlying AD pathology. However, when seizure frequency decreased following antiseizure treatment, CSF Aβ levels declined in absolute concentration while the Aβ42/40 ratio remained reduced. This dissociation may be attributable, at least in part, to incomplete seizure control, as seizures did not completely resolve despite treatment, and may also reflect an underlying abnormality of amyloid metabolism, including concomitant early AD pathology. The persistently reduced Aβ42/40 ratio therefore cannot be explained solely by seizure activity. Concurrently, cognitive function improved, and amyloid PET performed at the age of 82 was assessed as visually negative. Based on these findings, the patient was considered to have LOEU as the primary etiology at that time, although concomitant AD pathology could not be entirely excluded. Accordingly, positive AD biomarkers alone should not be considered sufficient evidence that the clinical syndrome is primarily driven by AD pathology in patients with LOEU.

We therefore hypothesize that long-standing epileptic hyperexcitability may have contributed to increased activity-dependent Aβ production over many years, thereby facilitating the development of an underlying low-level amyloid process. The subsequent increase in amyloid PET signal during follow-up is more appropriately interpreted as progression of this pre-existing amyloid pathology rather than *de novo* plaque formation during the two-year interval.

During periods of inadequate seizure control, sustained neuronal hyperexcitability may have contributed to alterations in CSF Aβ dynamics, resulting in an abnormal CSF AD biomarker profile characterized by a reduced Aβ42/40 ratio. In contrast, suppression of neuronal hyperexcitability by perampanel may have contributed to reduced Aβ production, resulting in decreased CSF Aβ levels while the Aβ42/40 ratio remained reduced. When seizure frequency increased again at the age of 84, amyloid PET demonstrated cortical tracer uptake, which we interpreted as progression of the pre-existing low-level amyloid process rather than *de novo* plaque formation during the two-year interval. Acute perampanel administration has been reported to suppress β-cleavage of APP and reduce Aβ40 and Aβ42 levels in transgenic AD mouse models (Ueda et al., 2023), and the CSF findings in this patient are consistent with the hypothesis that perampanel may have contributed to reduced activity-dependent Aβ production via AMPA receptor inhibition, although this hypothesis remains speculative and a causal relationship cannot be established from a single case observation.

The association between epilepsy and AD-related pathology has been recognized for over a century, beginning with the description of senile plaque-like lesions in elderly patients with chronic epilepsy (Blocq and Marinesco, 1892). Neuropathological studies of resected brain tissue from patients with refractory temporal lobe epilepsy have since demonstrated heterogeneous AD-like changes, including age-dependent amyloid deposition, widespread phosphorylated tau pathology, or atypical Aβ and tau immunoreactivity without fully developed senile plaques or neurofibrillary tangles (Gourmaud et al., 2020). These findings suggest that chronic epileptic activity and neuronal hyperexcitability may promote AD-like molecular and biomarker alterations without necessarily representing irreversible AD pathology. This concept is consistent with the present case, in which CSF AD core biomarkers were positive despite fluctuating cognitive function and delayed amyloid PET positivity.

The relationship between epilepsy and CSF AD core biomarkers has attracted increasing attention in recent years. Previous studies have reported that patients with LOEU may exhibit decreased CSF Aβ42 levels and increased p-tau concentrations (Costa et al., 2016; Fernandes et al., 2022). Moreover, a substantial proportion of cognitively normal individuals with LOEU have been shown to have reduced CSF Aβ42 levels at baseline, and a subset subsequently develop AD during follow-up (Costa et al., 2019). These findings suggest that LOEU may include cases of preclinical AD. LOEU accompanied by cognitive impairment and reduced CSF Aβ42 levels has therefore been considered to represent a population at particularly high risk for AD (Romoli et al., 2021).

In contrast, the present case demonstrates that LOEU with cognitive impairment and positive CSF AD core biomarkers does not necessarily indicate irreversible neurodegenerative disease. Despite the positive CSF AD biomarker profile, characterized by a reduced CSF Aβ42/40 ratio and elevated p-tau levels, in the setting of cognitive decline, the patient exhibited treatment-responsive and reversible cognitive impairment. This observation suggests that LOEU with positive CSF AD core biomarkers may encompass heterogenous pathophysiological mechanisms, including potentially reversible epilepsy-related cognitive dysfunction. Consequently, reliance on CSF AD core biomarkers alone may lead to misdiagnosis of AD in certain cases (Figure 3).

**Figure 3:**
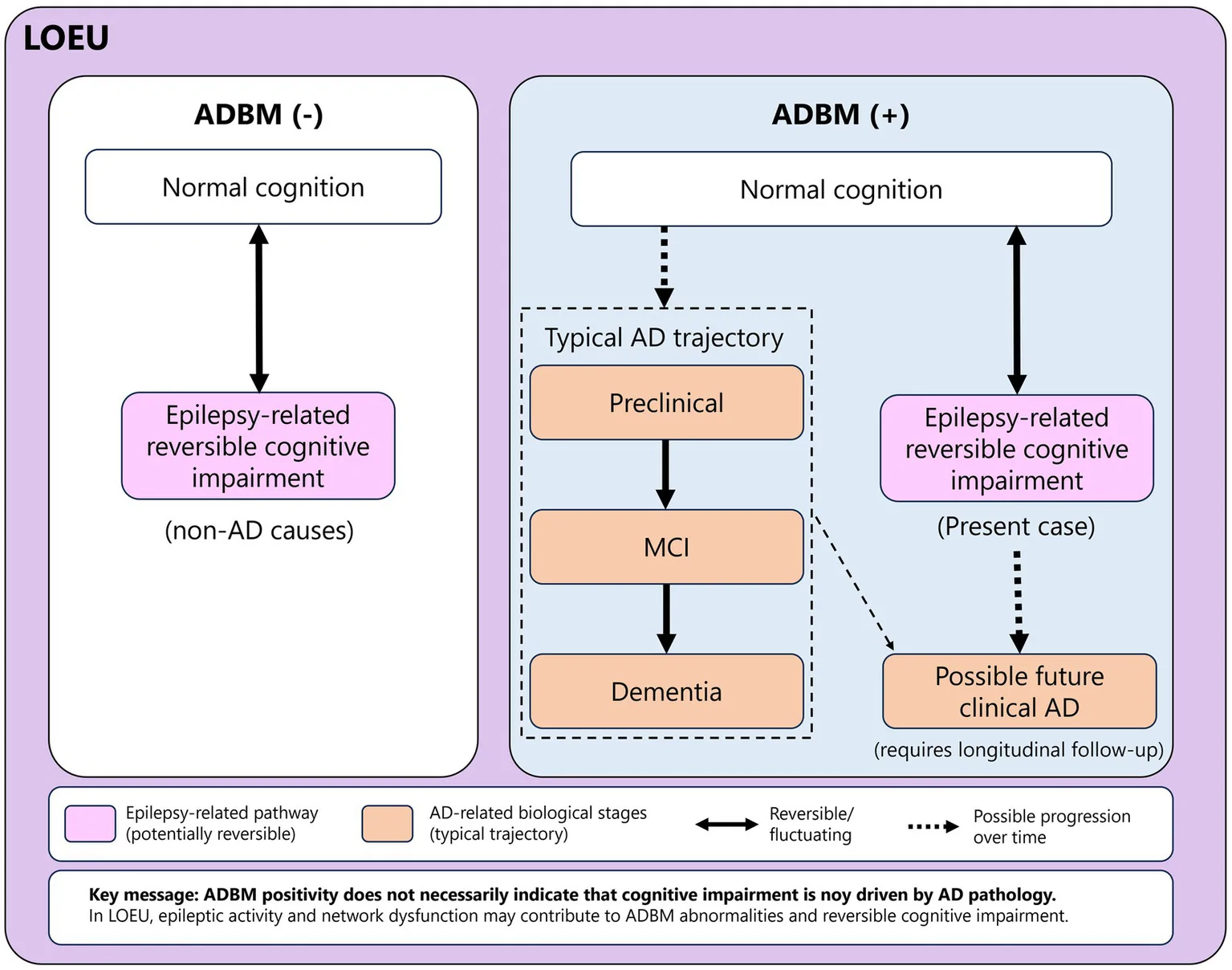
Relationship between late-onset epilepsy with unknown etiology (LOEU) and Alzheimer’s disease biomarkers (ADBM). Some patients with LOEU who are positive for ADBM are cognitively unimpaired and may therefore correspond to preclinical AD, and the clinical course has generally been assumed to progress unidirectionally from normal cognition through MCI to clinical AD. However, the present case demonstrates that cognitive impairment in an ADBM-positive patient with LOEU may be reversible, although slowly progressive AD pathology cannot be excluded. This indicates that cognitive impairment in ADBM-positive LOEU should not be equated with progression toward clinical AD, as it may be reversible, and underscores the need for caution to avoid premature diagnosis of AD. LOEU, late-onset epilepsy of unknown etiology; AD, Alzheimer’s disease; ADBM, Alzheimer’s disease biomarkers; MCI, mild cognitive impairment.

Several limitations should be acknowledged. This report describes a single case, which limits the generalizability of the findings. In addition, the precise relationship between seizure frequency and CSF AD core biomarker dynamics remains incompletely understood (Banote et al., 2022). The first lumbar puncture was performed at age 75; therefore, CSF data at disease onset are not available, and pre-epilepsy biomarker levels remain unknown. CSF sampling was limited to select time points throughout the follow-up period, and biomarker data at the time of the final relapse were not obtained. Although all CSF Aβ samples were analysed simultaneously in a single analytical batch using the same analytical platform, some degree of analytical variability cannot be completely excluded. A further limitation is that slowly progressive AD pathology cannot be excluded in this case. Although the relative stability of CSF p-tau and the Aβ42/40 ratio over a certain period (from age 78 to 80) supported epileptic activity as a primary contributor to the biomarker profile, the subsequent positivity on amyloid PET suggests that concomitant early AD pathology may have been present. As such, this case may reflect an interaction between epileptic activity and evolving AD pathology, rather than a clear dichotomy between the two. The persistent elevation of CSF p-tau throughout the clinical course warrants careful interpretation. CSF p-tau elevation has been reported in the context of epilepsy, potentially reflecting seizure-related neuronal injury or chronic network stress rather than AD-specific tau pathology (Gourmaud et al., 2020; Tai et al., 2016; Canet et al., 2022; Farhan et al., 2025). However, the failure of p-tau to normalize following improved seizure control raises the possibility that AD-related tau biology may have contributed, at least in part, to the persistently elevated p-tau levels in this case. Whether p-tau elevation reflects ongoing AD pathology, chronic neuronal injury secondary to long-standing epileptic activity, or a combination of these mechanisms remains uncertain. Regarding the interpretation of CSF Aβ changes following perampanel introduction, several confounding factors should also be acknowledged. The observed reduction in absolute CSF Aβ40 and Aβ42 concentrations occurred in the context of concurrent medication changes, including gabapentin withdrawal and ongoing treatment with valproate and levetiracetam, which may have independently influenced CSF Aβ levels. Furthermore, the reduction in seizure burden itself may have contributed to decreased activity-dependent Aβ production, independent of any direct pharmacological effect of perampanel, and changes in sleep architecture or sedation associated with antiseizure medication adjustments could also have affected Aβ metabolism. Therefore, a direct causal relationship between perampanel and the observed reduction in CSF Aβ levels cannot be established from this single case observation, and the proposed mechanism should be regarded as hypothesis-generating. Serum autoimmune antibody testing, routine inflammatory CSF studies, and epilepsy-protocol MRI were not performed, as the clinical presentation and negative CSF autoimmune antibody panel were considered sufficient for diagnosis at the time. APOE genotyping was not performed, as it was not covered by national health insurance in Japan at that time, limiting the assessment of genetic susceptibility to AD pathology in this case. Tau PET and plasma biomarkers, which may have provided additional insights into the temporal evolution of AD-related pathology, were not performed. The sensitivity of 24-h EEG for detecting subclinical epileptic activity is inherently limited, and interictal epileptiform discharges may have been underestimated. The timing of cognitive assessments relative to recent seizures was not systematically documented; therefore, transient post-ictal effects on neuropsychological test performance cannot be excluded. Repeated administration of the MMSE and Wechsler Memory Scale-Revised Logical Memory test may also have resulted in practice effects, potentially contributing to the observed improvement in cognitive performance. Finally, and most fundamentally, the absence of neuropathological confirmation means that the underlying biological diagnosis remains presumptive. As this is a single-case report, the findings should be regarded as hypothesis-generating and cannot be generalized without further studies.

Taken together, the overall longitudinal biomarker trajectory—including persistently positive CSF AD biomarkers and progression from visually negative to visually positive amyloid PET—is compatible with evolving AD pathology. At the same time, the marked decline in absolute CSF Aβ40 and Aβ42 levels during improved seizure control suggests that chronic epileptic activity may also influence biomarker dynamics. These observations indicate that evolving AD pathology and seizure-related biological effects are not mutually exclusive and may coexist throughout the disease course.

Nevertheless, this case highlights the importance of long-term, multimodal assessment—including cognitive evaluation, CSF AD core biomarker analysis, EEG, and both FDG- and amyloid PET imaging—in elucidating the pathophysiological mechanisms underlying LOEU.

LOEU may encompass both preclinical AD and potentially treatable forms of dementia, both of which can present positive CSF AD core biomarkers. Therefore, careful interpretation of CSF AD core biomarkers within the appropriate clinical and electrophysiological context is essential, particularly in the current era of anti-amyloid antibody therapies.

## Patient perspective

The patient reported progressive memory difficulties and increasing anxiety related to recurrent seizures, which interfered with daily activities and independence. During periods of poor seizure control, he experienced marked cognitive decline and frustration in social settings. Following optimization of antiseizure therapy, particularly after the introduction of perampanel, he noticed improvement in both seizure frequency and cognitive function, which allowed him to regain confidence in daily life. The patient expressed relief regarding the reversibility of his symptoms and emphasized the importance of long-term follow-up and individualized treatment.

## Data Availability

The datasets presented in this article are not readily available due to the nature of this single-patient case report, as the datasets consist of detailed longitudinal clinical, neuroimaging, electro physiological, and biomarker data. Sharing these data, even in anonymized form, carries a risk of patient re-identification. Therefore, the datasets are not available for external sharing. All relevant data supporting the findings of this study are included within the manuscript. Requests to access the datasets should be directed to AK, akuzuya@kuhp.kyoto-u.ac.jp.
