# Supplementary material for: Case Report: Longitudinal evaluation of cerebrospinal fluid Alzheimer’s disease core biomarkers in a case of refractory late-onset epilepsy of unknown etiology
Source: Front Aging Neurosci. 2026 Oct 12;18:1807027. doi: 10.3389/fnagi.2026.1807027 (PMC13725761; doi:10.3389/fnagi.2026.1807027)
Supplement: Supplementary file 1 [file Table_1.DOCX]

**Supplemental Table 1**

WMS-R scores are reported as index scores (mean = 100, SD = 15). WAIS-Ⅲ scores are reported as standard scores (mean = 100, SD = 15) for IQ and index scores. The Word Fluency Test included both category and phonemic conditions. For the category conditions, participants were asked to generate as many items as possible belonging to the categories of vegetables and animals. For the phonemic condition, participants were asked to generate as many words as possible beginning with the Japanese mora "ka" (romanized as "Ka"), a standard phonemic stimulus in Japanese neuropsychological assessment.

Abbreviations: MMSE, Mini-Mental State Examination; WMS-R, Wechsler Memory Scale-Revised; WAIS, Wechsler Adult Intelligence Scale; FSIQ, Full Scale IQ; VIQ, Verbal IQ; VC, Verbal Comprehension; WM, Working Memory; PIQ, Performance IQ; PO, Perceptual Organization; PS, Processing Speed; ADAS-J cog (11-item Japanese version), Alzheimer’s Disease Assessment Scale–Japanese cognitive subscale; FAB, Frontal Assessment Battery; WCST, Wisconsin Card Sorting Test; CA, Categories Achieved; PEN, Perseverative Errors; DMS, Difficulties in Maintaining Set; CDR, Clinical Dementia Rating; CDR-SB, Clinical Dementia Rating-Sum of Boxes.

|  | | **Age** | | | | | | | | | | | |
| --- | --- | --- | --- | --- | --- | --- | --- | --- | --- | --- | --- | --- | --- |
| **Neuropsychological Test** | | **70** | **71** | **73** | **74** | **75** | **78** | **79** | **80** | **81** | **82** | **83** | **84** |
| MMSE | | 29/30 | 27/30 | 27/30 | 26/30 | 28/30 | 23/30 | 26/30 | 27/30 | 29/30 | 28/30 | 28/30 | 23/30 |
| Logical Memory Test Ⅰ | | 23/50 | 30/50 | 21/50 | 19/50 | 12/50 | 16/50 | 11/50 | 17/50 | 12/50 | 17/50 | N/A | N/A |
| Logical Memory Test Ⅱ | | 11/50 | 28/50 | 14/50 | 6/50 | 8/50 | 9/50 | 3/50 | 9/50 | 13/50 | 1/50 | N/A | N/A |
| WMS-R | General Memory | N/A | N/A | N/A | 97 | 90 | 94 | N/A | 93 | N/A | N/A | N/A | N/A |
|  | Verbal Memory |  |  |  | 97 | 88 | 93 |  | 95 |  |  |  |  |
|  | Attention/Concentration |  |  |  | 80 | 89 | 78 |  | 84 |  |  |  |  |
|  | Visual Memory |  |  |  | 100 | 100 | 98 |  | 93 |  |  |  |  |
|  | Delayed Recall |  |  |  | 84 | 93 | 78 |  | 82 |  |  |  |  |
| WAIS-Ⅲ | FSIQ | N/A | N/A | N/A | 95 | 104 | 91 | N/A | N/A | N/A | N/A | N/A | N/A |
|  | VIQ |  |  |  | 91 | 96 | 90 |  |  |  |  |  |  |
|  | VC |  |  |  | 95 | 90 | 92 |  |  |  |  |  |  |
|  | WM |  |  |  | 83 | 102 | 92 |  |  |  |  |  |  |
|  | PIQ |  |  |  | 101 | 113 | 95 |  |  |  |  |  |  |
|  | PO |  |  |  | 95 | 110 | 95 |  |  |  |  |  |  |
|  | PS |  |  |  | 97 | 110 | 110 |  |  |  |  |  |  |
| ADAS-J cog | | 6.4/70 | 6.6/70 | 4.7/70 | 8.7/70 | 13.4/70 | 11.0/70 | 7.6/70 | 10.0/70 | 14.0/70 | 7.7/70 | N/A | N/A |
| FAB | | N/A | 14/18 | 15/18 | 15/18 | 11/18 | 13/18 | N/A | N/A | N/A | N/A | 14/18 | N/A |
| WCST | 1^st^ Administration | N/A | N/A | N/A | CA 1  PEN 15  DME 1 | CA 2  PEN 10  DME 4 | CA 1  PEN 8  DME 6 | N/A | N/A | N/A | N/A | N/A | N/A |
|  | 2^nd^ Administration |  |  |  | CA 1  PEN 25  DMS 1 | CA 0  PEN 3  DMS 6 | CA 1  PEN 2  DMS 6 |  |  |  |  |  |  |
| Clock Drawing Test | | N/A | N/A | N/A | N/A | N/A | N/A | N/A | N/A | 9/10 | 7/10 | N/A | N/A |
| Trail Making Test-J A | | N/A | N/A | N/A | N/A | N/A | N/A | 77s | 71s | 39s | 47s | N/A | N/A |
| Trail Making Test-J B | | N/A | N/A | N/A | N/A | N/A | N/A | 148s | 182s | 141s | 171s | N/A | N/A |
| Block Test  (WAIS Ⅳ) | Raw Score | N/A | N/A | N/A | N/A | N/A | N/A | 36/66 | 36/66 | N/A | N/A | N/A | N/A |
|  | Scaled Score |  |  |  |  |  |  | 11 | 11 |  |  |  |  |
| Word Fluency Test | | N/A | N/A | N/A | N/A | N/A | N/A | Vegetable 4  Animal 12  Ka 10 | Vegetable 6  Animal 10  Ka 10 | Vegetable 4  Animal 7  Ka 8 | Vegetable 8  Animal 7  Ka 8 | N/A | N/A |
| CDR Global | | N/A | N/A | N/A | N/A | N/A | N/A | N/A | N/A | N/A | N/A | 0.5 | 0.5 |
| CDR-SB | | N/A | N/A | N/A | N/A | N/A | N/A | N/A | N/A | N/A | N/A | 2 | 1.5 |
