# Supplementary material for: Case Report: Longitudinal evaluation of cerebrospinal fluid Alzheimer’s disease core biomarkers in a case of refractory late-onset epilepsy of unknown etiology
Source: Front Aging Neurosci. 2026 Oct 12;18:1807027. doi: 10.3389/fnagi.2026.1807027 (PMC13725761; doi:10.3389/fnagi.2026.1807027)
Supplement: Supplementary file 2 [file Table_2.docx]

**Supplemental Table 2**

Regional standardized uptake value ratios (SUVr) of amyloid PET at ages 82 and 84 years were calculated using four reference regions (whole cerebellum, pons, cerebellar gray matter, and whole cerebellum plus brainstem).

Abbreviations: PET, positron emission tomography; SUVr, standardized uptake value ratios; WC, whole cerebellum; CG, cerebellar gray matter; WCB, whole cerebellum plus brainstem; L, left; R, right.

|  | **Region** | **SUVr (WC)** | **SUVr (Pons)** | **SUVr (CG)** | **SUVr (WCB)** |
| --- | --- | --- | --- | --- | --- |
| **Age 82** | L-Frontal lobe | 0.90795647 | 0.52674102 | 1.16198788 | 0.81754698 |
|  | R-Frontal lobe | 0.9757732 | 0.56608416 | 1.24877862 | 0.87861088 |
|  | L-Parietal lobe | 0.92175173 | 0.53474419 | 1.17964282 | 0.82996858 |
|  | R-Parietal lobe | 0.99414577 | 0.5767428 | 1.27229154 | 0.895154 |
|  | L-Temporal lobe | 1.00721746 | 0.5843262 | 1.28902048 | 0.90692409 |
|  | R-Temporal lobe | 1.13559514 | 0.65880311 | 1.45331614 | 1.0225186 |
|  | L-Occipital lobe | 1.0674489 | 0.61926881 | 1.36610368 | 0.96115799 |
|  | R-Occipital lobe | 1.06362056 | 0.61704784 | 1.36120424 | 0.95771086 |
|  | L-Posterior cingulate | 1.01484107 | 0.58874896 | 1.29877705 | 0.91378858 |
|  | R-Posterior cingulate | 1.068287 | 0.61975503 | 1.36717628 | 0.96191264 |
|  | L-Precuneus | 1.0433197 | 0.60527052 | 1.33522353 | 0.93943146 |
|  | R-Precuneus | 1.09654824 | 0.63615048 | 1.40334455 | 0.98735978 |
|  | L-Basal ganglia | 1.15489455 | 0.66999945 | 1.4780152 | 1.03989627 |
|  | R-Basal ganglia | 1.24065269 | 0.7197511 | 1.58776707 | 1.11711507 |
|  | L-Cerebellum | 0.90306659 | 0.52390421 | 1.15572988 | 0.813144 |
|  | R-Cerebellum | 0.90860018 | 0.52711446 | 1.16281168 | 0.81812659 |
| **Age 84** | L-Frontal lobe | 0.980030567 | 0.561761356 | 1.274242587 | 0.87921473 |
|  | R-Frontal lobe | 1.062239882 | 0.608884393 | 1.381131712 | 0.952967165 |
|  | L-Parietal lobe | 0.957100228 | 0.548617502 | 1.2444284 | 0.858643237 |
|  | R-Parietal lobe | 1.057666619 | 0.606262961 | 1.375185523 | 0.948864354 |
|  | L-Temporal lobe | 1.045699943 | 0.599403567 | 1.359626368 | 0.938128691 |
|  | R-Temporal lobe | 1.20435795 | 0.690347605 | 1.565914616 | 1.080465534 |
|  | L-Occipital lobe | 1.071261543 | 0.614055681 | 1.392861739 | 0.961060767 |
|  | R-Occipital lobe | 1.092238529 | 0.626079857 | 1.420136163 | 0.979879849 |
|  | L-Posterior cingulate | 1.034049291 | 0.59272532 | 1.344478109 | 0.927676543 |
|  | R-Posterior cingulate | 1.10886928 | 0.635612736 | 1.441759581 | 0.994799792 |
|  | L-Precuneus | 1.090758757 | 0.62523164 | 1.418212152 | 0.978552301 |
|  | R-Precuneus | 1.163010599 | 0.666646973 | 1.512154502 | 1.043371589 |
|  | L-Basal ganglia | 1.206805318 | 0.691750456 | 1.569096701 | 1.082661141 |
|  | R-Basal ganglia | 1.281149455 | 0.73436511 | 1.665759467 | 1.14935749 |
|  | L-Cerebellum | 0.902809524 | 0.517497637 | 1.173839247 | 0.809937423 |
|  | R-Cerebellum | 0.913658765 | 0.523716509 | 1.187945507 | 0.819670602 |
